# Therapeutic Notes

**Published:** 1899-03

**Authors:** W. J. Martin

**Affiliations:** Kankakee, Ill.


### THERAPEUTIC NOTES.

UNDER THE DIRECTION OF W. J. MARTIN, M.D.C.,
KANKAKEE, ILL.

THERAPEUTICS OF THE NEWER SILVER SALTS.—The search for the ideal antiseptic—one that will be a reliable germicide for surgical-wound dressings and at the same time free from local irritation and toxic absorbition when used for this purpose and also in those grave constitutional diseases of the animal organism due to the ravages of bacteria or their toxic ptomaïns, in which the remedy could be introduced directly into the blood-stream by the intravenous method—has been carried on with the utmost perseverance and vigor by physiological chemists since Lister made his epoch-making researches on the life-history of bacteria. As a result of these exhaustive biochemic researches in the domain of medicine and chemistry we have almost daily brought to notice some new chemical compound or derivative for which great therapeutic properties are claimed by the originators. Many of these new remedies, or, rather, new combinations of old drugs, possess but little therapeutic value, and, after a short, meteoric career, drop into the realm of obscurity; others, again, possess intrinsic therapeutic value, and some become important additions to the armamentarium of the physician.

Among the old chemical agents which have been submitted to the keen scrutiny of the physiological chemists in their search for the ideal antiseptic has been silver and its various salts. This element has of late yielded to chemistry many new compounds, and quite recently several new salts have been obtained from it. Some of these new silver-salts possess decided therapeutic value, according to the latest reports current in medical literature. The latest of these, and to which our attention has been but recently called, is known as "soluble metallic silver." It is claimed to be "of extreme value in the treatment of the pure or mixed infections caused by the staphylococcus, the streptococcus, and other bacilli." It is in the treatment of acute processes—as phlegmons, lymphangiectasis, and lymphadenitis, septicæmia, phlegmonous erysipelas, etc.—that argentum "crede," or soluble metallic silver, has been quite extensively used in surgery and in skin-diseases by the medical profession in Germany. Judging from the favorable recommendations of the users, this silver-salt is of decided value both in the form of a solution and by inunction in the form of an ointment. It is said to be entirely non-toxic and unirritating in its action.

In veterinary practice Dickerhoff has used the drug in four cases of phlegmonous swelling occurring about the head, legs, throat, and abdomen of horses, with decided benefit in every case. In one of these cases, a mare suffering from morbus maculosis following attacks of influenza, the head and throat were so greatly swollen as to prevent the ingestion of either food or water. Dieckerhoff gave this mare, intravenously, 0.5 gramme (7½ grains) of the soluble silver in 50 grammes (14 drachms) of distilled water every two hours until 2½ grammes (37½ grains) of the soluble silver had been given in this manner, with the result that the animal fully recovered in a few days. The drug can also be administered subcutaneously with an ordinary hypodermatic syringe, and in this manner can be injected directly into the swollen tissues.

For treating the various forms of skin-diseases Dieckerhoff gives the following formula of soluble metallic silver: Argent. colloidalii, 15.0; ceræ, 10.0; adipis sulli., 90.0; ætheric benzoti, 10.0. Sig. For inunction. This is usually made on the sound side of the body—*i. e.*, on the opposite side from the diseased area.

The soluble silver may also be used in pill form by mixing with sugar of milk, glycerin, and water. In surgical cases—such as open wounds, recent or old, fungous or tubercular, affecting the bones or the soft parts—these pills may be placed deeply into the diseased parts. Soluble silver may also be used in a 1 or 2 per cent. solution, and thus used as a spray to wounds.

Soluble metallic silver occurs in the form of small, hard lumps, with a greenish metallic lustre. It can be triturated into a fine powder. When dissolved in water it forms a brownish-colored liquid, and after being filtered no precipitate takes place.

The price of this drug is three dollars per ounce.

*Lactate of Silver.* This is also a new silver-salt, and, being very soluble in water, it is claimed by its originators to be an efficient antiseptic and germicide in the strength of 1 : 500 to 1 : 1000 parts of water. It is indicated in the treatment of phagedenic ulcers, fistulas, abscesses, simple sores, or wounds.

*Argonin.* Argonin is a combination of silver and casein, obtained by mixing a solution of casein sodium with silver nitrate and precipitating with alcohol. It is a fine, white powder, readily soluble in warm water, and contains 4.25 per cent. of silver. The aqueous solution may be mixed with chlorides, alkalies, or serous fluid without precipitation. Consequently when brought into contact with albuminous secretion the silver is not precipitated in an inert form, and, owing to the absence of a free alkali or acid, no caustic or irritant effect upon the tissues is possible, nor is pain occasioned by its use.

Reports of experiments made to determine its action upon bacteria show that it possesses marked antiseptic properties. A 10 per cent. solution in water is the proper strength to use argonin as an antiseptic. It is largely employed at the present time in human practice in the treatment of gonorrhœa.

FOR COUGH.—For the dry and irritating cough so often met with in young horses suffering from influenza, the following combination will give relief: ℞.—Tr. Acon. rad., ♏. 20; tr. belladonna folia, ℥j; comp. tinct. opium, ℥j.—M. Sig.—May be given at one dose and repeated three times a day. It is best given in a few ounces of sweetened water.

IN India large areas of swampy country are being planted by the government with sunflowers, the growth of which, it is claimed, is more beneficial in eradicating malarial fever than even the eucalyptus tree.

---

TOXIC PTOMAÏNS OF PRESERVED MEATS. Van Eimenglin (*Journ. de Par.*) states that the toxic ptomaïns sometimes found in preserved meats, hams, game, pies, etc., are due to the presence of a specific organism—*bacillus bolulinus.* The soluble ptomaïn it secretes, called bolulin by the author, is stated to be so intensely toxic that one-thousandth part of a milligrammme killed a rabbit in twenty-four hours. Fortunately, this ptomaïn is destroyed at a temperature of 60° to 70° C. and the bacillus which produces it at 85° C., so that thorough cooking will remove all danger in the case of smoked or salted meats.

In this connection it is interesting to report that a recent issue of the *Journal of the American Medical Association* records cases of ptomaïn-poisoning resulting from supposed fresh ham. Within a few hours after eating a number of persons became ill and suffered with intense pain in the abdominal region, accompanied by nausea and vomiting. A day or two later another family bought ham from the same grocer, and after the two elder sons had eaten of it for lunch they were shortly after likewise afflicted. The following morning two daughters and a younger son ate of the ham, and were afterward seized with similar symptoms. All these cases, however, terminated favorably.—*Bull. Pharmacy.*

